# Significance of Anti-TPO as an Early Predictive Marker in Thyroid Disease

**DOI:** 10.1155/2019/1684074

**Published:** 2019-07-28

**Authors:** Thushani Siriwardhane, Karthik Krishna, Vinodh Ranganathan, Vasanth Jayaraman, Tianhao Wang, Kang Bei, Sarah Ashman, Karenah Rajasekaran, John J. Rajasekaran, Hari Krishnamurthy

**Affiliations:** ^1^Vibrant America LLC., San Carlos, CA, USA; ^2^Vibrant Sciences LLC., San Carlos, CA, USA

## Abstract

Even though most thyroid subjects are undiagnosed due to nonspecific symptoms, universal screening for thyroid disease is not recommended for the general population. In this study, our motive is to showcase the early appearance of thyroid autoantibody, anti-TPO, prior to the onset of thyroid hormone disruption; hence the addition of anti-TPO in conjunction with traditional thyroid markers TSH and FT4 would aid to reduce the long-term morbidity and associated health concerns. Here, a total of 4581 subjects were tested multiple times for TSH, FT4, anti-TPO, and anti-Tg and followed up for 2 years. We streamlined our subjects into two groups, A1 (euthyroid at first visit, but converted to subclinical/overt hypothyroidism in follow-up visits) and A2 (euthyroid at first visit, but converted to hyperthyroidism in follow-up visits). According to our results, 73% of hypothyroid subjects (from group A1) and 68.6% of hyperthyroid subjects (from group A2) had anti-TPO 252 (±33) and 277 (±151) days prior to the onset of the thyroid dysfunction, respectively. Both subclinical/overt hypothyroidism and hyperthyroidism showed a significantly higher percentage of subjects who had anti-TPO prior to the onset of thyroid dysfunction compared to the combined control group. However, there was no significant difference in the subjects who had anti-Tg earlier than the control group. Further assessment showed that only anti-TPO could be used as a standalone marker but not anti-Tg. Our results showcase that anti-TPO appear prior to the onset of thyroid hormone dysfunction; hence testing anti-TPO in conjunction with TSH would greatly aid to identify potentially risk individuals and prevent long-term morbidity.

## 1. Introduction

Thyroid dysfunction, which is defined as a broad spectrum of disorders related to the thyroid gland, has an enormous effect on human health. Approximately 20 million people are suffering from any form of thyroid disease in the United States [1]. The prevalence of thyroid dysfunction varies in each population that can be attributed to geographic/environmental factors, ethnicity, age, sex, etc. Functional thyroid disease is mainly divided into hypothyroidism (underactive thyroid) and hyperthyroidism (overactive thyroid) that is further subdivided into overt and subclinical disease. It is estimated that approximately 4.6% of the US population are suffering from hypothyroidism (0.3% clinical and 4.3% subclinical) and 1.3% from hyperthyroidism (0.5% clinical and 0.7% subclinical) [2]. Many people with thyroid disease go undiagnosed since symptoms develop gradually and are not very specific. Even though screening for thyroid disease seems appropriate, universal screening has not been endorsed unanimously due to lack of clinical trials that establish the benefits of subsequent therapy [3–5].

Although universal screening is not recommended, some studies show that early treatment, especially for subclinical hypothyroidism, could be beneficial for prevention of developing to overt hypothyroidism and its attendant morbidity [6], and prevention of future heart diseases by correcting serum lipid profiles [7]. Some studies highlight that thyroid disease is associated with aberrant lipid profiles and cardiac abnormalities [8, 9]; hence early diagnosis and treatment would possibly eliminate the need for expensive lipid-lowering therapies. In addition, early diagnosis and treatment would decrease the cost of evaluating and treating nonspecific symptoms. However, frequent screening advocates for high-risk groups such as high-risk pregnant women since undetected subclinical hypothyroidism and the presence of thyroid autoantibodies during pregnancy may adversely affect the survival of the fetus and are associated with hypertension and toxemia [10–12]. However, studies have demonstrated the cost-effectiveness of universal screening for pregnant women compared with screening only for high-risk pregnant women with no screening [13]. Not only pregnant women, but also women who are in their child bearing age should be tested and treated early since data suggest that subclinical hypothyroidism is associated with ovulatory dysfunction and infertility [14].

Autoimmune thyroid disease is the most common form of thyroid dysfunction causing several forms of thyroiditis ranging from hypothyroidism (Hashimoto's thyroiditis) and hyperthyroidism (Graves's Disease). Thyroid autoimmunity is characterized by thyroid autoantibodies, especially anti-TPO and anti-Tg. In recent years, autoantibodies have shown valuable results as early diagnostic markers in many diseases such as cancer, rheumatoid arthritis, and celiac disease [15–17]. Unfortunately, thyroid autoantibodies are routinely checked only after any abnormality is found in thyroid hormones, especially TSH and FT4. But their occurrence even before the TSH marker, which is the primary marker, has not been appreciated.

In this study, our focus is to showcase the early appearance of thyroid autoantibodies in euthyroid subjects which later developed into hypothyroidism and hyperthyroidism. Our findings showcase that measuring anti-TPO in conjunction with TSH and FT4 would be beneficial in identifying euthyroid subjects with potential risk of developing thyroid disease and, thus, be helpful for close monitoring, frequent follow-ups, and early treatment decisions to prevent long-term morbidity and associated diseases such as cardiovascular disease [18–21].

## 2. Materials and Methods

### 2.1. Patient Selection and Study Design

The eligible study population comprised 4581 subjects who were tested multiple times at Vibrant America Clinical Laboratory for minimum of four thyroid disease markers (free T4 hormone (FT4), TSH, anti-TPO, anti-Tg) between April 2016 and April 2018 (2 years). This retrospective analysis was completed using deidentified clinical data and test results, hence was exempted from formal ethical reviews by Western IRB (Washington, USA). Subjects were categorized as below for analysis purposes.

Group A1: Euthyroid at their first visit but converted to subclinical/overt hypothyroidism in later visits.

Group A2: Euthyroid at their first visit but converted to subclinical/overt hyperthyroidism in later visits.

The demographics of the subjects are listed in Table 1.

### 2.2. Reference Ranges for Thyroid Markers

Thyroid hormone reference ranges are subject to the lab where the test is performed. In this study, we followed the reference ranges that majority of the labs used. The reference range of hormones and autoantibody levels in a healthy control used in this study are shown in Table 2. Subclinical hypothyroidism, subclinical hyperthyroidism, overt hypothyroidism, and overt hyperthyroidism were attributed using these ranges.

The categorization of thyroid disease status by evaluating TSH and FT4 levels used in this study is shown in Table 3.

### 2.3. TSH, FT4, Anti-TPO, and Anti-Tg Tests

TSH, FT4, anti-TPO, and anti-Tg were measured using the commercial Roche e601 Analyzer (Roche Diagnostics, Indianapolis, IN, USA), according to the manufacturer's recommendations. The reagents were purchased from Roche Diagnostics (Indianapolis, IN, USA). Human serum specimens were used for electrochemiluminescence immunoassay on Elecsys immunoassay analyzers. In brief, the Elecsys TSH assay employed monoclonal antibodies specifically directed against human TSH. The antibodies labeled with a ruthenium complex consist of a chimeric construct from human and mouse-specific components. As a result, interfering effects due to HAMA (human anti-mouse antibodies) were largely eliminated.

For the Elecsys FT4 test, the determination of free thyroxine was made with the aid of a specific anti-T4 antibody labeled with a ruthenium complex. The quantity of antibody used was so small (equivalent to approx. 1-2% of the total T4 content of a normal serum sample) that the equilibrium between bound and unbound T4 remained virtually unaffected.

Recombinant antigens and polyclonal anti-TPO antibodies were used in the Elecsys anti-TPO assay, whereas human antigen and monoclonal human anti-Tg antibodies were used in Elecsys anti-Tg assay.

### 2.4. Patient and Public Involvement

This study does not include any patient or public involvement and the study is based on retrospective analysis of deidentified laboratory data, hence was exempted from formal ethical reviews by Western Institutional Review Board (WIRB).

### 2.5. Statistical Analysis

The processing and analysis of clinical data from deidentified subjects were performed via Java for Windows version 1.8.161. Data were expressed as mean ± standard deviation (SD) with a Gaussian distribution. Multiple logistic regression analysis was used to evaluate the presence of clinical variables. P < 0.05 was considered significant.

## 3. Results

### 3.1. Anti-TPO as an Early Predictive Marker

We sought to assess the early appearance of anti-TPO and anti-Tg markers in subjects with autoimmune thyroid disease. The subjects were divided into two groups: group A1 consist of subjects who were euthyroid at their first visit but converted to subclinical/overt hypothyroidism in their follow-up visits and group A2 consist of subjects who were euthyroid at their first visit but converted to subclinical/overt hyperthyroidism in their follow-up visits. The percentage distribution of physicians reported ICD-10-CM (International Classification of Diseases, Tenth Revision, Clinical Modification) codes were used to provide clinical information on group A1 and group A2 subjects. As expected, 25.7% and 46.6% of hypothyroid and hyperthyroid subjects, respectively, complained of vitamin D deficiency (ICD-10-CM-E559) and 25.0% hypothyroid and 56.8% hyperthyroid subjects complained of other fatigues (ICD-10-CM-R5383); both are nonspecific symptoms. Similarly, 23.0% hypothyroid subjects and 46.6% hyperthyroid subjects have listed hypothyroidism (ICD-10-CM-E039) as the cause for testing. The percentage distribution of the full spectrum of ICD-10-CM codes of group A1 and group A2 subjects is listed in Supplementary Table S1.

Group A1 had a total of 152 subjects who developed hypothyroidism, and 127 of them converted to subclinical hypothyroidism (83.5%) and 25 converted to overt hypothyroidism (16.5%) during their 2-year follow-up visits. Group A2 had a total of 118 subjects who developed hyperthyroidism and 95 of them converted to subclinical hyperthyroidism (80.5%) and 23 converted to overt hyperthyroidism (19.5%) during the 2-year follow-up visits.

**Figure 1:**
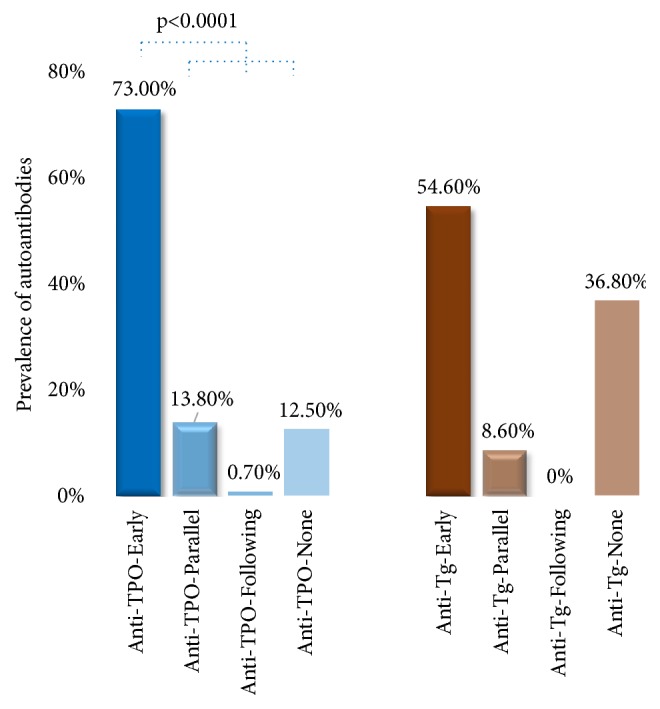
The prevalence of anti-TPO (anti-TPO-Early, blue) and anti-Tg (anti-Tg-Early, orange) prior to the onset of subclinical/overt hypothyroidism compared to the combined control groups. Anti-TPO combined control group (blue) consists of anti-TPO-Parallel, anti-TPO-Following, and anti-TPO-None. Anti-Tg combined control group (orange) consists of anti-Tg-Parallel, anti-Tg-Following, and anti-Tg-None.

Figure 1 shows the frequency of subjects in group A1 who had anti-TPO and anti-Tg earlier than the onset of subclinical/overt hypothyroidism during the 2 years of follow-up assessment. We assessed the number of subjects who had anti-TPO prior to the onset of subclinical/overt hypothyroidism and compared them to the control group (subjects with anti-TPO parallel, following, or never to the onset of subclinical/overt hypothyroidism). As shown in Figure 1, 111/152 (73.0%) subjects had anti-TPO prior to the onset of subclinical/overt hypothyroidism, 21/152 (13.8%) had anti-TPO parallel to the onset of subclinical/overt hypothyroidism, 1/152 (0.7%) had anti-TPO following the onset of subclinical/overt hypothyroidism, and 19/152 (12.5%) had no anti-TPO. The later 3 groups (parallel, following, and never) were combined to create the control group (41/152 (27.0%)) to compare the significance of anti-TPO occurring prior to the onset of subclinical/overt hypothyroidism. The incidence of anti-TPO positivity seen earlier than the onset of subclinical/overt hypothyroidism in group A1 was significantly higher (p<0.0001) than the combined control group and anti-TPO was positive in an average of 252(±33) days ahead of the onset of subclinical/overt hypothyroidism. The same group was assessed for the occurrence of anti-Tg. As shown in Figure 1, 83/152 (54.6%) subjects had anti-Tg prior to the onset of subclinical/overt hypothyroidism, 13/152 (8.6%) had anti-Tg parallel to the onset of subclinical/overt hypothyroidism, 0/42 (0%) had anti-Tg following the onset of subclinical/overt hypothyroidism, and 56/152 (36.8%) had no anti-Tg. The incidence of anti-Tg positivity seen earlier than the onset of subclinical/overt hypothyroidism was not significant.

**Figure 2:**
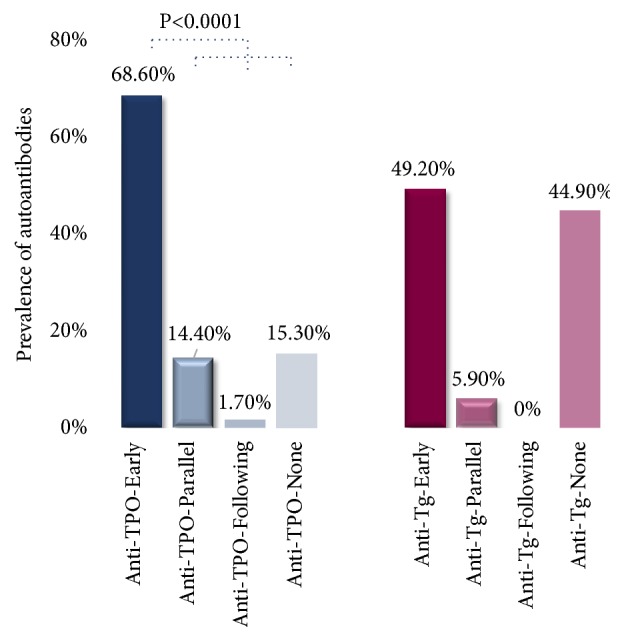
The prevalence of anti-TPO (anti-TPO-Early, blue) and anti-Tg (anti-Tg-Early, burgundy) prior to the onset of subclinical/overt hyperthyroidism compared to the combined control groups. Anti-TPO combined control group (blue) consists of anti-TPO-Parallel, anti-TPO-Following, and anti-TPO-None. Anti-Tg combined control group (burgundy) consists of anti-Tg-Parallel, anti-Tg-Following, and anti-Tg-None.

**Figure 3:**
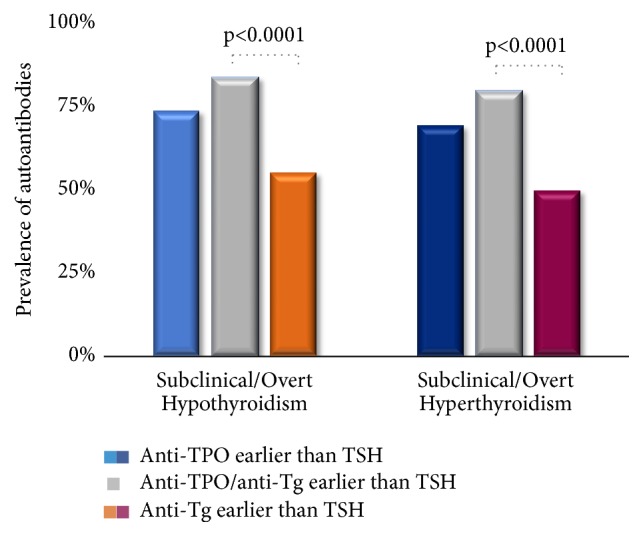
Comparison of the prevalence of anti-TPO (light blue/dark blue), anti-Tg (orange/burgundy) and combined anti-TPO or anti-Tg (grey) in subjects converted to subclinical/overt hypothyroidism (group A1) and hyperthyroidism (group A2).

The same assessment was conducted for group A2 subjects. As shown in Figure 2, 81/118 (68.6%) subjects had anti-TPO prior to the onset of subclinical/overt hyperthyroidism, 17/118 (14.4%) had anti-TPO parallel to the onset of subclinical/overt hyperthyroidism, 2/118 (1.7%) had anti-TPO following the onset of subclinical/overt hyperthyroidism, and 18/118 (15.3%) had no anti-TPO. The incidence of anti-TPO positivity seen earlier than the onset of subclinical/overt hyperthyroidism was significantly higher (p<0.0001) than the combined control group and the anti-TPO was positive in an average of 277 (±151) days ahead of the onset of subclinical/overt hyperthyroidism. The same group was assessed for the occurrence of anti-Tg. As shown in Figure 3, 58/152 (49.2%) subjects had anti-Tg prior to the onset of subclinical/overt hyperthyroidism, 7/152 (5.9%) had anti-Tg parallel to the onset of subclinical/overt hyperthyroidism, 0/42 (0%) had anti-Tg following the onset of subclinical/overt hyperthyroidism, and 53/152 (44.9%) had no anti-Tg. The incidence of anti-Tg positivity seen earlier than the onset of subclinical/overt hyperthyroidism was not significant.

Next, we sought to evaluate each antibody's ability to perform as a standalone test. We assessed the subjects in groups A1 and A2. In group A1, we assessed three categories and compared them to each other: subjects who had either anti-TPO or anti-Tg prior to the onset of subclinical/overt hypothyroidism, subjects who had anti-TPO prior to the onset of subclinical/overt hypothyroidism, and subjects who had anti-Tg prior to the onset of subclinical/overt hypothyroidism. A similar analysis was performed for group A2. As shown in Figure 3, 126/152 (82.9%) subjects in group A1 had either anti-TPO or anti-Tg prior to the onset of subclinical/overt hypothyroidism compared to 111/152 (73.0%, p= 0.0522) with anti-TPO and 83/152 (54.6%, p<0.0001) with anti-Tg. Similarly, 93/118 (78.8%) subjects in group A2 had either anti-TPO or anti-Tg prior to the onset of subclinical/overt hyperthyroidism compared to 81/118 (68.6%, p= 0.1033) with anti-TPO and 58/118 (49.2%, p<0.0001) with anti-Tg. No significant difference was found when anti-TPO was measured alone compared to the combined measurement of either anti-TPO or anti-Tg occurrence prior to the onset of subclinical/overt hypothyroidism and hyperthyroidism. But a significantly low number of subjects were reported when anti-Tg was considered alone compared to the combined assessment of either anti-TPO or anti-Tg measurement.

## 4. Discussion

The goal of this study is to showcase the early appearance of anti-TPO autoantibodies, prior to the onset of biochemical changes in thyroid hormones, TSH and FT4. Measuring anti-TPO along with TSH and FT4 benefits as a predictive marker in diagnosing euthyroid subjects who might be at risk for potential thyroid dysfunctions. Many individuals may not seek medical attention due to the gradual development of symptoms or due to nonspecific symptoms. This was clearly evidenced in evaluating clinical symptoms reported by the physicians of these subjects, in terms of ICD-10-CM codes. The ICD-10-CM is an internationally recognized system, listed by the World Health Organization (WHO) and used by physicians and other healthcare providers to report their diagnosis, symptoms, and procedures as a unique alphanumeric code specific for each disease, disorder, injury, infection, and symptom. It details necessary information for diagnostic specificity and morbidity classification in the USA and across the world. We have calculated and ranked the percentage distribution of ICD-10-CM codes that the physicians have reported based on patient symptoms, behavior, and history. The top ranked ICD-10-CM code was vitamin D deficiency (ICD-10-CM-E559) followed by fatigue (ICD-10-CM-R5383) and hypothyroidism (ICD-10-CM-E039). Thyroid disease and vitamin D deficiency share some common symptoms such as fatigue and hair loss. This may be attributed to the high number of reported ICD-10-CM codes for vitamin D deficiency and fatigue. Moreover, significantly low levels of vitamin D were documented in patients with autoimmune thyroid disease [22]. However, we have seen that 46.6% of physicians' have reported ICD-10-CM code which is for hypothyroidism for their patients but their serum biochemistry reported hyperthyroidism. Hypothyroidism and hyperthyroidism have common nonspecific symptoms; hence it is possible that most of these symptoms could lead the physician to report as hypothyroidism (ICD-10-CM-E039) given that hypothyroidism is more common than hyperthyroidism.

Treatment or frequent testing is not warranted in subjects whose TSH levels are not derailed or borderline. In this study, we showed that presence of anti-TPO even without any disruptions in thyroid hormone levels indicates a possible risk of developing thyroid dysfunction. According to our results, 73% of hypothyroid subjects and 68.6% of hyperthyroid subjects had anti-TPO 252 (±33) and 277 (±151) days prior to the onset of the thyroid dysfunction, respectively. We compared our results with a combined control group which consist of subjects who developed anti-TPO at the same time as the thyroid dysfunction, later than the onset of thyroid dysfunction, and had no anti-TPO. Both subclinical/overt hypothyroidism and hyperthyroidism showed high percentage of subjects who had anti-TPO prior to the onset of thyroid dysfunction compared to the combined control group. However, there was no significant difference in the subjects who had anti-Tg earlier than the control group. This is expected, since anti-Tg is a well-established marker in differentiated thyroid cancer (DTC) diagnosis and is considered a less specific marker in thyroid disease compared to anti-TPO [23]. Even though our study was limited to 2 years, our results correlate with a study performed by Hutfless et al. that showed the preexistence of anti-TPO and anti-Tg autoantibodies 7 years prior to the concise diagnosis of Hashimoto's (66% and 57% for anti-TPO and anti-Tg, respectively) and Graves' disease (57% and 47% for anti-TPO and anti-Tg, respectively) for 174 patients [24].

Since anti-TPO antibodies were dominant to anti-Tg antibodies in all our results, we analyzed whether these two markers can be used as standalone markers. Our final set of results on group A1 and group A2 showed that there was no significant difference when anti-TPO was performed alone compared to the combination of anti-TPO or anti-Tg assessment. Hence, there was no significant effect from the addition of anti-Tg marker to the anti-TPO performance and thus can be used as a standalone marker. But when the positivity of anti-Tg was assessed alone and compared to the combined anti-TPO or anti-Tg assessment, the combined group of anti-TPO or anti-Tg showed significantly higher results than anti-Tg alone. Hence, it clearly verified that anti-Tg is not a favorable standalone marker and if performed should always combine with anti-TPO.

In this study, our motive was to showcase the benefit of adding anti-TPO, as a first-tier test in combination with TSH and FT4; hence subjects with normal TSH and elevated autoantibodies would not be neglected but referred for frequent follow-ups. Addition of one test could potentially save expenditure on long-term diseases such as overt thyroid disease and its attended morbidities, associated dysfunctions in reproductive health, especially in women at child bearing age, and cardiovascular diseases. However, a large study should be performed to evaluate the other variables that can affect thyroid disease status such as current thyroid treatments and smoking habits. In conclusion, our results showed that thyroid autoantibodies precede subclinical/overt hypothyroidism and hyperthyroidism. Hence, it may be beneficial to consider testing for anti-TPO in conjunction with the primary thyroid markers, TSH and FT4, to prevent long-term morbidity.

## Figures and Tables

**Table 1:** Demographics of the subjects studied.

	A1	A2
Number	152	118
Age (X±SD)	52±17	50±12
Gender	124F/28M	106F/12M

**Table 2:** Reference ranges for thyroid markers studied.

Marker	Reference Range
TSH	0.3-4.2 mIU/L
FT4	0.9-1.7 ng/dL
Anti-TPO	<9.0 IU/mL
Anti-Tg	<4.0 IU/mL

**Table 3:** Thyroid disease categorization.

Disease Condition	TSH	FT4
Subclinical hypothyroidism	> 4.2 mIU/L	0.9-1.7 ng/dL
Subclinical hyperthyroidism	< 0.3 mIU/L	0.9-1.7 ng/dL
Overt hypothyroidism	> 4.2 mIU/L	< 0.9 ng/dL
Overt hyperthyroidism	< 0.3 mIU/L	> 1.7 ng/dL

## Data Availability

The datasets used and/or analyzed during the current study are available from the corresponding author on reasonable request.

## Supplementary Materials

Supplementary Materials Table S1: physician reported ICD-10-CM codes distribution for hypothyroid and hyperthyroid subjects.
