# Supplementary material for: Significance of Anti-TPO as an Early Predictive Marker in Thyroid Disease
Source: Autoimmune Dis. 2019 Jul 28;2019:1684074. doi: 10.1155/2019/1684074 (PMC6699358; doi:10.1155/2019/1684074)
Supplement: Supplementary Materials — Table S1: physician reported ICD-10-CM codes distribution for hypothyroid and hyperthyroid subjects. [file 1684074.f1.zip › SI-Thyroid Autoantibodies as Predictive Markers_AD_2828351.docx]

**Supplementary Materials**

**Significance of Anti-TPO as an Early Predictive Marker in Thyroid Disease**

Thushani Siriwardhane^1^*, Karthik Krishna^2^, Vinodh Ranganathan^2^, Vasanth Jayaraman^2^, Tianhao Wang^2^, Kang Bei^2^, Sarah Ashman^1^, Karenah Rajasekaran^2^, John J. Rajasekaran^2^, Hari Krishnamurthy^2^*

*^1^Vibrant America LLC., San Carlos, CA, USA*

*^2^Vibrant Sciences LLC., San Carlos, CA, USA*

**Running title:** Anti-TPO as a Predictive Marker in Thyroid Disease

**Address correspondence to:** Thushani Siriwardhane, PhD, Vibrant America LLC., 1360 Bayport Ave, San Carlos, CA 94070, USA. Phone: 650-508-8262, Fax: 650-508-8262. E-mail: [thushanis@vibrant-america.com](mailto:thushanis@vibrant-america.com); Hari Krishnamurthy, Vibrant Sciences LLC., 1021 Howard Avenue, Suite B, San Carlos, CA 94070, USA. Phone: 1-866-364-0963, Fax: 1-650-508-8262. E-mail: [hari@vibrant](mailto:hari@vibrant)sci.com

Table S1. Physician reported ICD-10-CM codes distribution for hypothyroid and hyperthyroid subjects.

| **ICD-10-CM** | **Description** | **Hypothyroid Subjects (%)** | **Hyperthyroid Subjects (%)** |
| --- | --- | --- | --- |
| E559 | Vitamin D deficiency, unspecified | 25.7 | 46.6 |
| R5383 | Other fatigue | 25.0 | 56.8 |
| E039 | Hypothyroidism, unspecified | 23.0 | 46.6 |
| E782 | Mixed hyperlipidemia | 14.5 | 18.6 |
| E349 | Endocrine disorder, unspecified | 11.8 | 19.5 |
| I10 | Essential (primary) hypertension | 10.5 | 7.6 |
| M2550 | Pain in unspecified joint | 9.9 | 16.9 |
| E785 | Hyperlipidemia, unspecified | 7.9 | 12.7 |
| R799 | Abnormal finding of blood chemistry, unspecified | 7.2 | 14.4 |
| N951 | Menopausal and female climacteric states | 7.2 | 18.6 |
| R7989 | Other specified abnormal findings of blood chemistry | 6.6 | 5.1 |
| Z79899 | Other long term (current) drug therapy | 6.6 | 11.0 |
| Z0000 | Encounter for general adult medical examination without abnormal findings | 5.9 | 7.6 |
| D539 | Nutritional anemia, unspecified | 4.6 | 15.3 |
| E291 | Testicular hypofunction | 4.6 | 4.2 |
| F419 | Anxiety disorder, unspecified | 3.9 | 9.3 |
| R197 | Diarrhea, unspecified | 3.9 | 1.7 |
| E079 | Disorder of thyroid, unspecified | 3.9 | 2.5 |
| E119 | Type 2 diabetes mellitus without complications | 3.9 | 2.5 |
| R5382 | Chronic fatigue, unspecified | 3.3 | 3.4 |
| E063 | Autoimmune thyroiditis | 3.3 | 4.2 |
| R5381 | Other malaise | 2.6 | 5.9 |
| E7211 | Homocystinuria | 2.6 | 2.5 |
| K210 | Gastro-esophageal reflux disease with esophagitis | 2.6 | 1.7 |
| R638 | Other symptoms and signs concerning food and fluid intake | 2.6 | 9.3 |
| R6882 | Decreased libido | 2.6 | 7.6 |
| R6889 | Other general symptoms and signs | 2.6 | 0 |
| K5900 | Constipation, unspecified | 2.0 | 7.6 |
| Z8249 | Family history of ischemic heart disease and other diseases of the circulatory system | 2.0 | 0.8 |
| E8881 | Metabolic syndrome | 2.0 | 7.6 |
| R7301 | Impaired fasting glucose | 2.0 | 1.7 |
| R7982 | Elevated C-reactive protein (CRP) | 2.0 | 2.5 |
| G4700 | Insomnia, unspecified | 2.0 | 3.4 |
| R109 | Unspecified abdominal pain | 2.0 | 0.8 |
| D509 | Iron deficiency anemia, unspecified | 2.0 | 2.5 |
| R635 | Abnormal weight gain | 2.0 | 2.5 |
| Z13220 | Encounter for screening for lipoid disorders | 2.0 | 0 |
| R634 | Abnormal weight loss | 2.0 | 2.5 |
| E639 | Nutritional deficiency, unspecified | 1.3 | 2.5 |
| E279 | Disorder of adrenal gland, unspecified | 1.3 | 0 |
| I2510 | Atherosclerotic heart disease of native coronary artery without angina pectoris | 1.3 | 1.7 |
| I4891 | Unspecified atrial fibrillation | 1.3 | 0.8 |
| J311 | Chronic nasopharyngitis | 1.3 | 0.8 |
| J449 | Chronic obstructive pulmonary disease, unspecified | 1.3 | 0 |
| K589 | Irritable bowel syndrome without diarrhea | 1.3 | 2.5 |
| K909 | Intestinal malabsorption, unspecified | 1.3 | 5.9 |
| N959 | Unspecified menopausal and perimenopausal disorder | 1.3 | 11 |
| R0602 | Shortness of breath | 1.3 | 3.4 |
| R748 | Abnormal levels of other serum enzymes | 1.3 | 0 |
| Z13228 | Encounter for screening for other metabolic disorders | 1.3 | 0.8 |
| D508 | Other iron deficiency anemias | 1.3 | 0.8 |
| E1165 | Type 2 diabetes mellitus with hyperglycemia | 1.3 | 2.5 |
| E669 | Obesity, unspecified | 1.3 | 1.7 |
| E881 | Lipodystrophy, not elsewhere classified | 1.3 | 0.8 |
| Z131 | Encounter for screening for diabetes mellitus | 1.3 | 0 |
| Z136 | Encounter for screening for cardiovascular disorders | 1.3 | 0 |
| E282 | Polycystic ovarian syndrome | 1.3 | 0 |
| E538 | Deficiency of other specified B group vitamins | 1.3 | 2.5 |
| G894 | Chronic pain syndrome | 1.3 | 0 |
| I119 | Hypertensive heart disease without heart failure | 1.3 | 0 |
| N529 | Male erectile dysfunction, unspecified | 1.3 | 0.8 |
| R4584 | Anhedonia | 1.3 | 0 |
| Z79890 | Hormone replacement therapy (postmenopausal) | 1.3 | 0 |
| Z833 | Family history of diabetes mellitus | 1.3 | 0.8 |
| E038 | Other specified hypothyroidism | 0.7 | 0.8 |
| E663 | Overweight | 0.7 | 0.8 |
| F329 | Major depressive disorder, single episode, unspecified | 0.7 | 2.5 |
| K219 | Gastro-esophageal reflux disease without esophagitis | 0.7 | 1.7 |
| K580 | Irritable bowel syndrome with diarrhea | 0.7 | 1.7 |
| R531 | Weakness | 0.7 | 2.5 |
| R739 | Hyperglycemia, unspecified | 0.7 | 1.7 |
| C8590 | Non-Hodgkin lymphoma, unspecified, unspecified site | 0.7 | 0 |
| D803 | Selective deficiency of immunoglobulin G [IgG] subclasses | 0.7 | 0 |
| E035 | Myxedema coma | 0.7 | 0 |
| E1129 | Type 2 diabetes mellitus with other diabetic kidney complication | 0.7 | 0 |
| E289 | Ovarian dysfunction, unspecified | 0.7 | 0 |
| E83119 | Hemochromatosis, unspecified | 0.7 | 0 |
| G8929 | Other chronic pain | 0.7 | 0 |
| H6120 | Impacted cerumen, unspecified ear | 0.7 | 0 |
| I209 | Angina pectoris, unspecified | 0.7 | 0 |
| I270 | Primary pulmonary hypertension | 0.7 | 0 |
| I348 | Other nonrheumatic mitral valve disorders | 0.7 | 0 |
| K900 | Celiac disease | 0.7 | 0.8 |
| L659 | Nonscarring hair loss, unspecified | 0.7 | 0.8 |
| M545 | Low back pain | 0.7 | 0 |
| N958 | Other specified menopausal and perimenopausal disorders | 0.7 | 0 |
| O99352 | Diseases of the nervous system complicating pregnancy, second trimester | 0.7 | 0 |
| Q909 | Down syndrome, unspecified | 0.7 | 0 |
| R400 | Somnolence | 0.7 | 0 |
| R451 | Restlessness and agitation | 0.7 | 0 |
| R51 | Headache | 0.7 | 0 |
| R945 | Abnormal results of liver function studies | 0.7 | 1.7 |
| T7840XA | Allergy, unspecified, initial encounter | 0.7 | 2.5 |
| Z139 | Encounter for screening, unspecified | 0.7 | 0 |
| Z780 | Asymptomatic menopausal state | 0.7 | 0 |
| Z951 | Presence of aortocoronary bypass graft | 0.7 | 0 |
| Z95810 | Presence of automatic (implantable) cardiac defibrillator | 0.7 | 0 |
| A09 | Infectious gastroenteritis and colitis, unspecified | 0.7 | 0 |
| D649 | Anemia, unspecified | 0.7 | 1.7 |
| D721 | Eosinophilia | 0.7 | 0 |
| E0500 | Thyrotoxicosis with diffuse goiter without thyrotoxic crisis or storm | 0.7 | 0 |
| E0590 | Thyrotoxicosis, unspecified without thyrotoxic crisis or storm | 0.7 | 0.8 |
| E0781 | Sick-euthyroid syndrome | 0.7 | 0 |
| E569 | Vitamin deficiency, unspecified | 0.7 | 0 |
| E60 | Dietary zinc deficiency | 0.7 | 0 |
| E630 | Essential fatty acid [EFA] deficiency | 0.7 | 0 |
| E631 | Imbalance of constituents of food intake | 0.7 | 0 |
| E638 | Other specified nutritional deficiencies | 0.7 | 1.7 |
| E781 | Pure hyperglyceridemia | 0.7 | 0 |
| E784 | Other hyperlipidemia | 0.7 | 1.7 |
| E799 | Disorder of purine and pyrimidine metabolism, unspecified | 0.7 | 0 |
| F1099 | Alcohol use, unspecified with unspecified alcohol-induced disorder | 0.7 | 0 |
| F411 | Generalized anxiety disorder | 0.7 | 0 |
| F430 | Acute stress reaction | 0.7 | 0 |
| F4541 | Pain disorder exclusively related to psychological factors | 0.7 | 0 |
| F938 | Other childhood emotional disorders | 0.7 | 0 |
| G3184 | Mild cognitive impairment, so stated | 0.7 | 0 |
| G43719 | Chronic migraine without aura, intractable, without status migrainosus | 0.7 | 0 |
| G479 | Sleep disorder, unspecified | 0.7 | 1.7 |
| I428 | Other cardiomyopathies | 0.7 | 0 |
| I480 | Paroxysmal atrial fibrillation | 0.7 | 0 |
| I498 | Other specified cardiac arrhythmias | 0.7 | 0 |
| I509 | Heart failure, unspecified | 0.7 | 1.7 |
| I739 | Peripheral vascular disease, unspecified | 0.7 | 0 |
| I779 | Disorder of arteries and arterioles, unspecified | 0.7 | 0 |
| I83893 | Varicose veins of bilateral lower extremities with other complications | 0.7 | 0 |
| I872 | Venous insufficiency (chronic) (peripheral) | 0.7 | 0 |
| K259 | Gastric ulcer, unspecified as acute or chronic, without hemorrhage or perforation | 0.7 | 0 |
| K5190 | Ulcerative colitis, unspecified, without complications | 0.7 | 0 |
| K599 | Functional intestinal disorder, unspecified | 0.7 | 0 |
| K9049 | Malabsorption due to intolerance, not elsewhere classified | 0.7 | 0 |
| L209 | Atopic dermatitis, unspecified | 0.7 | 0 |
| L603 | Nail dystrophy | 0.7 | 0 |
| L709 | Acne, unspecified | 0.7 | 0 |
| M13169 | Monoarthritis, not elsewhere classified, unspecified knee | 0.7 | 0 |
| M5410 | Radiculopathy, site unspecified | 0.7 | 0 |
| M5489 | Other dorsalgia | 0.7 | 0.8 |
| M549 | Dorsalgia, unspecified | 0.7 | 0 |
| M797 | Fibromyalgia | 0.7 | 0 |
| N400 | Benign prostatic hyperplasia without lower urinary tract symptoms | 0.7 | 0 |
| N649 | Disorder of breast, unspecified | 0.7 | 0 |
| R000 | Tachycardia, unspecified | 0.7 | 0 |
| R001 | Bradycardia, unspecified | 0.7 | 0 |
| R011 | Cardiac murmur, unspecified | 0.7 | 0 |
| R062 | Wheezing | 0.7 | 0.8 |
| R21 | Rash and other nonspecific skin eruption | 0.7 | 0.8 |
| R351 | Nocturia | 0.7 | 0 |
| R41840 | Attention and concentration deficit | 0.7 | 0 |
| R42 | Dizziness and giddiness | 0.7 | 0.8 |
| R452 | Unhappiness | 0.7 | 0 |
| R453 | Demoralization and apathy | 0.7 | 0 |
| R4586 | Emotional lability | 0.7 | 0 |
| R600 | Localized edema | 0.7 | 0 |
| R61 | Generalized hyperhidrosis | 0.7 | 0 |
| R6259 | Other lack of expected normal physiological development in childhood | 0.7 | 0 |
| R6510 | Systemic inflammatory response syndrome (SIRS) of non-infectious origin without acute organ dysfunction | 0.7 | 0 |
| R7309 | Other abnormal glucose | 0.7 | 3.4 |
| R740 | Nonspecific elevation of levels of transaminase and lactic acid dehydrogenase [LDH] | 0.7 | 0 |
| R790 | Abnormal level of blood mineral | 0.7 | 0.8 |
| R891 | Abnormal level of hormones in specimens from other organs, systems and tissues | 0.7 | 0 |
| R947 | Abnormal results of other endocrine function studies | 0.7 | 0.8 |
| Z0001 | Encounter for general adult medical examination with abnormal findings | 0.7 | 0 |
| Z5181 | Encounter for therapeutic drug level monitoring | 0.7 | 0 |
| Z733 | Stress, not elsewhere classified | 0.7 | 0 |
| Z820 | Family history of epilepsy and other diseases of the nervous system | 0.7 | 0 |
| Z8349 | Family history of other endocrine, nutritional and metabolic diseases | 0.7 | 0 |
| Z8639 | Personal history of other endocrine, nutritional and metabolic disease | 0.7 | 0 |
| D518 | Other vitamin B12 deficiency anemias | 0 | 0.8 |
| D538 | Other specified nutritional anemias | 0 | 0.8 |
| D563 | Thalassemia minor | 0 | 0.8 |
| D682 | Hereditary deficiency of other clotting factors | 0 | 0.8 |
| E270 | Other adrenocortical overactivity | 0 | 2.5 |
| E2749 | Other adrenocortical insufficiency | 0 | 0.8 |
| E2839 | Other primary ovarian failure | 0 | 0.8 |
| E550 | Rickets, active | 0 | 0.8 |
| E617 | Deficiency of multiple nutrient elements | 0 | 1.7 |
| E876 | Hypokalemia | 0 | 0.8 |
| F0630 | Mood disorder due to known physiological condition, unspecified | 0 | 0.8 |
| H539 | Unspecified visual disturbance | 0 | 0.8 |
| J3089 | Other allergic rhinitis | 0 | 0.8 |
| J309 | Allergic rhinitis, unspecified | 0 | 0.8 |
| J410 | Simple chronic bronchitis | 0 | 0.8 |
| M069 | Rheumatoid arthritis, unspecified | 0 | 2.5 |
| M159 | Polyosteoarthritis, unspecified | 0 | 0.8 |
| M2410 | Other articular cartilage disorders, unspecified site | 0 | 0.8 |
| M542 | Cervicalgia | 0 | 0.8 |
| M791 | Myalgia | 0 | 0.8 |
| N912 | Amenorrhea, unspecified | 0 | 3.4 |
| N926 | Irregular menstruation, unspecified | 0 | 2.5 |
| N943 | Premenstrual tension syndrome | 0 | 0.8 |
| N946 | Dysmenorrhea, unspecified | 0 | 0.8 |
| N979 | Female infertility, unspecified | 0 | 0.8 |
| R202 | Paresthesia of skin | 0 | 0.8 |
| R457 | State of emotional shock and stress, unspecified | 0 | 0.8 |
| R718 | Other abnormality of red blood cells | 0 | 0.8 |
| R9720 | Elevated prostate specific antigen [PSA] | 0 | 0.8 |
| D899 | Disorder involving the immune mechanism, unspecified | 0 | 0.8 |
| E109 | Type 1 diabetes mellitus without complications | 0 | 0.8 |
| E230 | Hypopituitarism | 0 | 1.7 |
| E619 | Deficiency of nutrient element, unspecified | 0 | 1.7 |
| E662 | Morbid (severe) obesity with alveolar hypoventilation | 0 | 0.8 |
| E7800 | Pure hypercholesterolemia, unspecified | 0 | 0.8 |
| E8310 | Disorder of iron metabolism, unspecified | 0 | 0.8 |
| E860 | Dehydration | 0 | 1.7 |
| F17210 | Nicotine dependence, cigarettes, uncomplicated | 0 | 0.8 |
| F320 | Major depressive disorder, single episode, mild | 0 | 0.8 |
| H04129 | Dry eye syndrome of unspecified lacrimal gland | 0 | 0.8 |
| H8110 | Benign paroxysmal vertigo, unspecified ear | 0 | 0.8 |
| H9313 | Tinnitus, bilateral | 0 | 0.8 |
| I951 | Orthostatic hypotension | 0 | 0.8 |
| J321 | Chronic frontal sinusitis | 0 | 0.8 |
| J343 | Hypertrophy of nasal turbinates | 0 | 0.8 |
| K5222 | Food protein-induced enteropathy | 0 | 0.8 |
| M2500 | Hemarthrosis, unspecified joint | 0 | 0.8 |
| M810 | Age-related osteoporosis without current pathological fracture | 0 | 0.8 |
| N644 | Mastodynia | 0 | 0.8 |
| N923 | Ovulation bleeding | 0 | 0.8 |
| R002 | Palpitations | 0 | 1.7 |
| R110 | Nausea | 0 | 0.8 |
| R1310 | Dysphagia, unspecified | 0 | 0.8 |
| R140 | Abdominal distension (gaseous) | 0 | 0.8 |
| R143 | Flatulence | 0 | 0.8 |
| R760 | Raised antibody titer | 0 | 1.7 |
| R789 | Finding of unspecified substance, not normally found in blood | 0 | 0.8 |
| R946 | Abnormal results of thyroid function studies | 0 | 0.8 |
| Z793 | Long term (current) use of hormonal contraceptives | 0 | 0.8 |
| Z90710 | Acquired absence of both cervix and uterus | 0 | 0.8 |
