# Supplementary material for: Significance of Anti-TPO as an Early Predictive Marker in Thyroid Disease
Source: Autoimmune Dis. 2019 Jul 28;2019:1684074. doi: 10.1155/2019/1684074 (PMC6699358; doi:10.1155/2019/1684074)
Supplement: Supplementary Materials — Table S1: physician reported ICD-10-CM codes distribution for hypothyroid and hyperthyroid subjects. [file 1684074.f1.zip › SI-Thyroid Autoantibodies as Predictive Markers_AD_2828352.pdf]

Supplementary Materials

Significance of Anti-TPO as an Early Predictive Marker in Thyroid Disease

Thushani Siriwardhane[1]*, Karthik Krishna[2], Vinodh Ranganathan[2], Vasanth Jayaraman[2], Tianhao Wang[2], Kang Bei[2], Sarah Ashman[1], Karenah Rajasekaran[2], John J. Rajasekaran[2], Hari Krishnamurthy[2]*

[1]*Vibrant America LLC., San Carlos, CA, USA*

[2]*Vibrant Sciences LLC., San Carlos, CA, USA*

Running title: Anti-TPO as a Predictive Marker in Thyroid Disease

Address correspondence to: Thushani Siriwardhane, PhD, Vibrant America LLC., 1360 Bayport Ave, San Carlos, CA 94070, USA. Phone: 650-508-8262, Fax: 650-508-8262. E-mail: thushanis@vibrant-america.com; Hari Krishnamurthy, Vibrant Sciences LLC., 1021 Howard Avenue, Suite B, San Carlos, CA 94070, USA. Phone: 1-866-364-0963, Fax: 1-650-508-8262. E-mail: hari@vibrantsci.com

Table S1. Physician reported ICD-10-CM codes distribution for hypothyroid and hyperthyroid subjects.

ICD-10-CM	Description	Hypothyroid Subjects (%)	Hyperthyroid Subjects (%)
E559	Vitamin D deficiency, unspecified	25.7	46.6
R5383	Other fatigue	25.0	56.8
E039	Hypothyroidism, unspecified	23.0	46.6
E782	Mixed hyperlipidemia	14.5	18.6
E349	Endocrine disorder, unspecified	11.8	19.5
I10	Essential (primary) hypertension	10.5	7.6
M2550	Pain in unspecified joint	9.9	16.9
E785	Hyperlipidemia, unspecified	7.9	12.7
R799	Abnormal finding of blood chemistry, unspecified	7.2	14.4
N951	Menopausal and female climacteric states	7.2	18.6
R7989	Other specified abnormal findings of blood chemistry	6.6	5.1
Z79899	Other long term (current) drug therapy	6.6	11.0
Z0000	Encounter for general adult medical examination without abnormal findings	5.9	7.6
D539	Nutritional anemia, unspecified	4.6	15.3
E291	Testicular hypofunction	4.6	4.2
F419	Anxiety disorder, unspecified	3.9	9.3
R197	Diarrhea, unspecified	3.9	1.7
E079	Disorder of thyroid, unspecified	3.9	2.5
E119	Type 2 diabetes mellitus without complications	3.9	2.5
R5382	Chronic fatigue, unspecified	3.3	3.4
E063	Autoimmune thyroiditis	3.3	4.2
R5381	Other malaise	2.6	5.9
E7211	Homocystinuria	2.6	2.5
K210	Gastro-esophageal reflux disease with esophagitis	2.6	1.7
R638	Other symptoms and signs concerning food and fluid intake	2.6	9.3
R6882	Decreased libido	2.6	7.6
R6889	Other general symptoms and signs	2.6	0
K5900	Constipation, unspecified	2.0	7.6
Z8249	Family history of ischemic heart disease and other diseases of the circulatory system	2.0	0.8
E8881	Metabolic syndrome	2.0	7.6
R7301	Impaired fasting glucose	2.0	1.7
R7982	Elevated C-reactive protein (CRP)	2.0	2.5
G4700	Insomnia, unspecified	2.0	3.4
R109	Unspecified abdominal pain	2.0	0.8
D509	Iron deficiency anemia, unspecified	2.0	2.5

R635	Abnormal weight gain	2.0	2.5
Z13220	Encounter for screening for lipoid disorders	2.0	0
R634	Abnormal weight loss	2.0	2.5
E639	Nutritional deficiency, unspecified	1.3	2.5
E279	Disorder of adrenal gland, unspecified	1.3	0
I2510	Atherosclerotic heart disease of native coronary artery without angina pectoris	1.3	1.7
I4891	Unspecified atrial fibrillation	1.3	0.8
J311	Chronic nasopharyngitis	1.3	0.8
J449	Chronic obstructive pulmonary disease, unspecified	1.3	0
K589	Irritable bowel syndrome without diarrhea	1.3	2.5
K909	Intestinal malabsorption, unspecified	1.3	5.9
N959	Unspecified menopausal and perimenopausal disorder	1.3	11
R0602	Shortness of breath	1.3	3.4
R748	Abnormal levels of other serum enzymes	1.3	0
Z13228	Encounter for screening for other metabolic disorders	1.3	0.8
D508	Other iron deficiency anemias	1.3	0.8
E1165	Type 2 diabetes mellitus with hyperglycemia	1.3	2.5
E669	Obesity, unspecified	1.3	1.7
E881	Lipodystrophy, not elsewhere classified	1.3	0.8
Z131	Encounter for screening for diabetes mellitus	1.3	0
Z136	Encounter for screening for cardiovascular disorders	1.3	0
E282	Polycystic ovarian syndrome	1.3	0
E538	Deficiency of other specified B group vitamins	1.3	2.5
G894	Chronic pain syndrome	1.3	0
I119	Hypertensive heart disease without heart failure	1.3	0
N529	Male erectile dysfunction, unspecified	1.3	0.8
R4584	Anhedonia	1.3	0
Z79890	Hormone replacement therapy (postmenopausal)	1.3	0
Z833	Family history of diabetes mellitus	1.3	0.8
E038	Other specified hypothyroidism	0.7	0.8
E663	Overweight	0.7	0.8
F329	Major depressive disorder, single episode, unspecified	0.7	2.5
K219	Gastro-esophageal reflux disease without esophagitis	0.7	1.7
K580	Irritable bowel syndrome with diarrhea	0.7	1.7
R531	Weakness	0.7	2.5
R739	Hyperglycemia, unspecified	0.7	1.7
C8590	Non-Hodgkin lymphoma, unspecified, unspecified site	0.7	0
D803	Selective deficiency of immunoglobulin G [IgG] subclasses	0.7	0
E035	Myxedema coma	0.7	0
E1129	Type 2 diabetes mellitus with other diabetic kidney complication	0.7	0

E289	Ovarian dysfunction, unspecified	0.7	0
E83119	Hemochromatosis, unspecified	0.7	0
G8929	Other chronic pain	0.7	0
H6120	Impacted cerumen, unspecified ear	0.7	0
I209	Angina pectoris, unspecified	0.7	0
I270	Primary pulmonary hypertension	0.7	0
I348	Other nonrheumatic mitral valve disorders	0.7	0
K900	Celiac disease	0.7	0.8
L659	Nonscarring hair loss, unspecified	0.7	0.8
M545	Low back pain	0.7	0
N958	Other specified menopausal and perimenopausal disorders	0.7	0
O99352	Diseases of the nervous system complicating pregnancy, second trimester	0.7	0
Q909	Down syndrome, unspecified	0.7	0
R400	Somnolence	0.7	0
R451	Restlessness and agitation	0.7	0
R51	Headache	0.7	0
R945	Abnormal results of liver function studies	0.7	1.7
T7840XA	Allergy, unspecified, initial encounter	0.7	2.5
Z139	Encounter for screening, unspecified	0.7	0
Z780	Asymptomatic menopausal state	0.7	0
Z951	Presence of aortocoronary bypass graft	0.7	0
Z95810	Presence of automatic (implantable) cardiac defibrillator	0.7	0
A09	Infectious gastroenteritis and colitis, unspecified	0.7	0
D649	Anemia, unspecified	0.7	1.7
D721	Eosinophilia	0.7	0
E0500	Thyrotoxicosis with diffuse goiter without thyrotoxic crisis or storm	0.7	0
E0590	Thyrotoxicosis, unspecified without thyrotoxic crisis or storm	0.7	0.8
E0781	Sick-euthyroid syndrome	0.7	0
E569	Vitamin deficiency, unspecified	0.7	0
E60	Dietary zinc deficiency	0.7	0
E630	Essential fatty acid [EFA] deficiency	0.7	0
E631	Imbalance of constituents of food intake	0.7	0
E638	Other specified nutritional deficiencies	0.7	1.7
E781	Pure hyperglyceridemia	0.7	0
E784	Other hyperlipidemia	0.7	1.7
E799	Disorder of purine and pyrimidine metabolism, unspecified	0.7	0
F1099	Alcohol use, unspecified with unspecified alcohol-induced disorder	0.7	0

F411	Generalized anxiety disorder	0.7	0
F430	Acute stress reaction	0.7	0
F4541	Pain disorder exclusively related to psychological factors	0.7	0
F938	Other childhood emotional disorders	0.7	0
G3184	Mild cognitive impairment, so stated	0.7	0
G43719	Chronic migraine without aura, intractable, without status migrainosus	0.7	0
G479	Sleep disorder, unspecified	0.7	1.7
I428	Other cardiomyopathies	0.7	0
I480	Paroxysmal atrial fibrillation	0.7	0
I498	Other specified cardiac arrhythmias	0.7	0
I509	Heart failure, unspecified	0.7	1.7
I739	Peripheral vascular disease, unspecified	0.7	0
I779	Disorder of arteries and arterioles, unspecified	0.7	0
I83893	Varicose veins of bilateral lower extremities with other complications	0.7	0
I872	Venous insufficiency (chronic) (peripheral)	0.7	0
K259	Gastric ulcer, unspecified as acute or chronic, without hemorrhage or perforation	0.7	0
K5190	Ulcerative colitis, unspecified, without complications	0.7	0
K599	Functional intestinal disorder, unspecified	0.7	0
K9049	Malabsorption due to intolerance, not elsewhere classified	0.7	0
L209	Atopic dermatitis, unspecified	0.7	0
L603	Nail dystrophy	0.7	0
L709	Acne, unspecified	0.7	0
M13169	Monoarthritis, not elsewhere classified, unspecified knee	0.7	0
M5410	Radiculopathy, site unspecified	0.7	0
M5489	Other dorsalgia	0.7	0.8
M549	Dorsalgia, unspecified	0.7	0
M797	Fibromyalgia	0.7	0
N400	Benign prostatic hyperplasia without lower urinary tract symptoms	0.7	0
N649	Disorder of breast, unspecified	0.7	0
R000	Tachycardia, unspecified	0.7	0
R001	Bradycardia, unspecified	0.7	0
R011	Cardiac murmur, unspecified	0.7	0
R062	Wheezing	0.7	0.8
R21	Rash and other nonspecific skin eruption	0.7	0.8
R351	Nocturia	0.7	0
R41840	Attention and concentration deficit	0.7	0
R42	Dizziness and giddiness	0.7	0.8

R452	Unhappiness	0.7	0
R453	Demoralization and apathy	0.7	0
R4586	Emotional lability	0.7	0
R600	Localized edema	0.7	0
R61	Generalized hyperhidrosis	0.7	0
R6259	Other lack of expected normal physiological development in childhood	0.7	0
R6510	Systemic inflammatory response syndrome (SIRS) of non-infectious origin without acute organ dysfunction	0.7	0
R7309	Other abnormal glucose	0.7	3.4
R740	Nonspecific elevation of levels of transaminase and lactic acid dehydrogenase [LDH]	0.7	0
R790	Abnormal level of blood mineral	0.7	0.8
R891	Abnormal level of hormones in specimens from other organs, systems and tissues	0.7	0
R947	Abnormal results of other endocrine function studies	0.7	0.8
Z0001	Encounter for general adult medical examination with abnormal findings	0.7	0
Z5181	Encounter for therapeutic drug level monitoring	0.7	0
Z733	Stress, not elsewhere classified	0.7	0
Z820	Family history of epilepsy and other diseases of the nervous system	0.7	0
Z8349	Family history of other endocrine, nutritional and metabolic diseases	0.7	0
Z8639	Personal history of other endocrine, nutritional and metabolic disease	0.7	0
D518	Other vitamin B12 deficiency anemias	0	0.8
D538	Other specified nutritional anemias	0	0.8
D563	Thalassemia minor	0	0.8
D682	Hereditary deficiency of other clotting factors	0	0.8
E270	Other adrenocortical overactivity	0	2.5
E2749	Other adrenocortical insufficiency	0	0.8
E2839	Other primary ovarian failure	0	0.8
E550	Rickets, active	0	0.8
E617	Deficiency of multiple nutrient elements	0	1.7
E876	Hypokalemia	0	0.8
F0630	Mood disorder due to known physiological condition, unspecified	0	0.8
H539	Unspecified visual disturbance	0	0.8
J3089	Other allergic rhinitis	0	0.8
J309	Allergic rhinitis, unspecified	0	0.8
J410	Simple chronic bronchitis	0	0.8
M069	Rheumatoid arthritis, unspecified	0	2.5
M159	Polyosteoarthritis, unspecified	0	0.8

M2410	Other articular cartilage disorders, unspecified site	0	0.8
M542	Cervicalgia	0	0.8
M791	Myalgia	0	0.8
N912	Amenorrhea, unspecified	0	3.4
N926	Irregular menstruation, unspecified	0	2.5
N943	Premenstrual tension syndrome	0	0.8
N946	Dysmenorrhea, unspecified	0	0.8
N979	Female infertility, unspecified	0	0.8
R202	Paresthesia of skin	0	0.8
R457	State of emotional shock and stress, unspecified	0	0.8
R718	Other abnormality of red blood cells	0	0.8
R9720	Elevated prostate specific antigen [PSA]	0	0.8
D899	Disorder involving the immune mechanism, unspecified	0	0.8
E109	Type 1 diabetes mellitus without complications	0	0.8
E230	Hypopituitarism	0	1.7
E619	Deficiency of nutrient element, unspecified	0	1.7
E662	Morbid (severe) obesity with alveolar hypoventilation	0	0.8
E7800	Pure hypercholesterolemia, unspecified	0	0.8
E8310	Disorder of iron metabolism, unspecified	0	0.8
E860	Dehydration	0	1.7
F17210	Nicotine dependence, cigarettes, uncomplicated	0	0.8
F320	Major depressive disorder, single episode, mild	0	0.8
H04129	Dry eye syndrome of unspecified lacrimal gland	0	0.8
H8110	Benign paroxysmal vertigo, unspecified ear	0	0.8
H9313	Tinnitus, bilateral	0	0.8
I951	Orthostatic hypotension	0	0.8
J321	Chronic frontal sinusitis	0	0.8
J343	Hypertrophy of nasal turbinates	0	0.8
K5222	Food protein-induced enteropathy	0	0.8
M2500	Hemarthrosis, unspecified joint	0	0.8
M810	Age-related osteoporosis without current pathological fracture	0	0.8
N644	Mastodynia	0	0.8
N923	Ovulation bleeding	0	0.8
R002	Palpitations	0	1.7
R110	Nausea	0	0.8
R1310	Dysphagia, unspecified	0	0.8
R140	Abdominal distension (gaseous)	0	0.8
R143	Flatulence	0	0.8
R760	Raised antibody titer	0	1.7
R789	Finding of unspecified substance, not normally found in blood	0	0.8
R946	Abnormal results of thyroid function studies	0	0.8

Z793	Long term (current) use of hormonal contraceptives	0	0.8
Z90710	Acquired absence of both cervix and uterus	0	0.8
